# Gram-Negative Marine Bacteria: Structural Features of Lipopolysaccharides and Their Relevance for Economically Important Diseases

**DOI:** 10.3390/md12052485

**Published:** 2014-04-30

**Authors:** Muhammad Ayaz Anwar, Sangdun Choi

**Affiliations:** Department of Molecular Science and Technology, Ajou University, Suwon 443-749, Korea; E-Mail: ayaz@ajou.ac.kr

**Keywords:** Gram-negative bacteria, immune system, lipid A, lipopolysaccharide, marine organisms, TLR4

## Abstract

Gram-negative marine bacteria can thrive in harsh oceanic conditions, partly because of the structural diversity of the cell wall and its components, particularly lipopolysaccharide (LPS). LPS is composed of three main parts, an O-antigen, lipid A, and a core region, all of which display immense structural variations among different bacterial species. These components not only provide cell integrity but also elicit an immune response in the host, which ranges from other marine organisms to humans. Toll-like receptor 4 and its homologs are the dedicated receptors that detect LPS and trigger the immune system to respond, often causing a wide variety of inflammatory diseases and even death. This review describes the structural organization of selected LPSes and their association with economically important diseases in marine organisms. In addition, the potential therapeutic use of LPS as an immune adjuvant in different diseases is highlighted.

## 1. Introduction

The marine environment is enriched with bacteria that provide a fruitful source of natural substances [1,2,3], including antibiotics, antitumor agents, antitoxins, and enzymes, which have a broad range of applications. In recent years, much research attention has been devoted to isolating different metabolic intermediates and pathways as possible targets to treat diseases. Gram-negative bacteria are characterized by the presence of a unique cell wall component termed lipopolysaccharide (LPS), which is associated with substantial diseases in humans and marine organisms. LPS from non-pathogenic bacteria has been studied in detail to discover new therapeutics to combat lethal bacterial infections [4]. Currently, there is considerable research interest in determining the chemical features of LPS from Gram-negative bacteria thriving in marine environments.

**Figure 1:**
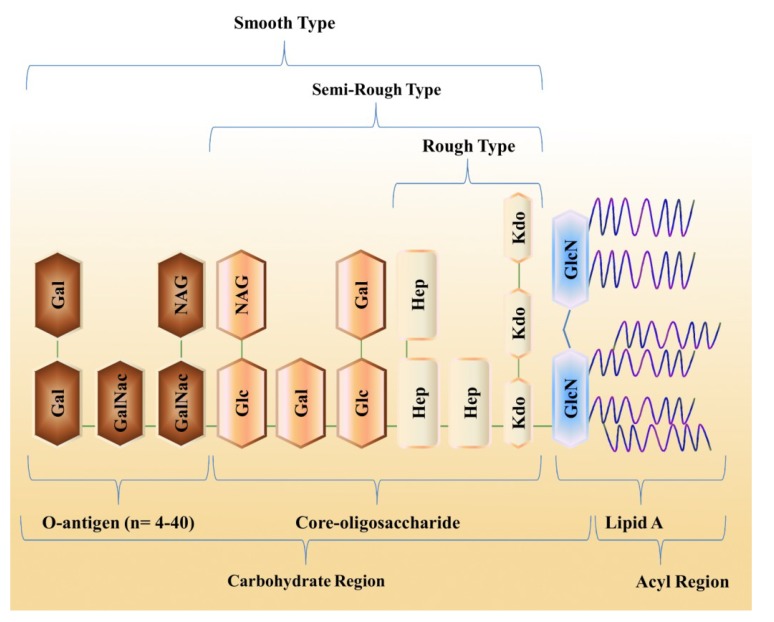
An overview of the complete LPS structure. LPS can be divided into three regions: O-antigen, core region, and lipid A. On the basis of the presence of the oligosaccharide, LPS can be classified into smooth, rough, and semi-rough types that pose different levels of threat during infection. Gal, galactose; GalNac, *N*-acetyl-galactosamine; Glc, glucose; GlcN, glucosamine; Hep, l-glycero-d-mannoheptose; Kdo, 3-deoxy-d-manno-2-octulonic acid; Man, mannose; NAG, *N*-acetyl-glucosamine.

Based on its appearance and composition, LPS can be divided into two types: smooth (S-type) and rough (R-type) (Figure 1). S-type LPS has additional components and is regarded as the most complete form that can be further subdivided into three parts in which (1) the O-antigenic outer region (generally a polymer of oligosaccharide units) is attached to (2) the hydrophobic membrane anchor, lipid A, by (3) a linker oligosaccharide known as the core. The core consists of 3-deoxy-d-manno-2-octulosonic acid (Kdo) and l-glycero-d-manno-heptose (l,d-Hep). Classically, lipid A is a dimer of d-glucosamine (d-GlcN) monomers linked in a β-1,6 fashion, to which fatty acid chains, typically 3-hydroxyalkanoic acids, are linked by amide or ester bonds. Anionic groups such as Kdo, phosphates, and other acidic residues are commonly present on the inner core region and lipid A. The polar residues in LPS, e.g., the polar head groups of phospholipids, are vital for the structural morphology and physiology of the outer membrane of bacteria [4].

In general, LPS is a conserved molecule with minor modifications; for instance, bacteria can be classified into different serotypes and serovars according to the degree of polymerization of S-type LPS [5,6]. The O-antigens are anionic, which facilitates the survival of these bacteria in oceanic environments. The O-antigen is appended to the core oligosaccharide (core-OS), which shows less variation and possesses particular saccharide units; however, some other groups in core-OS have also been reported in this region [7,8,9,10,11].

**Figure 2:**
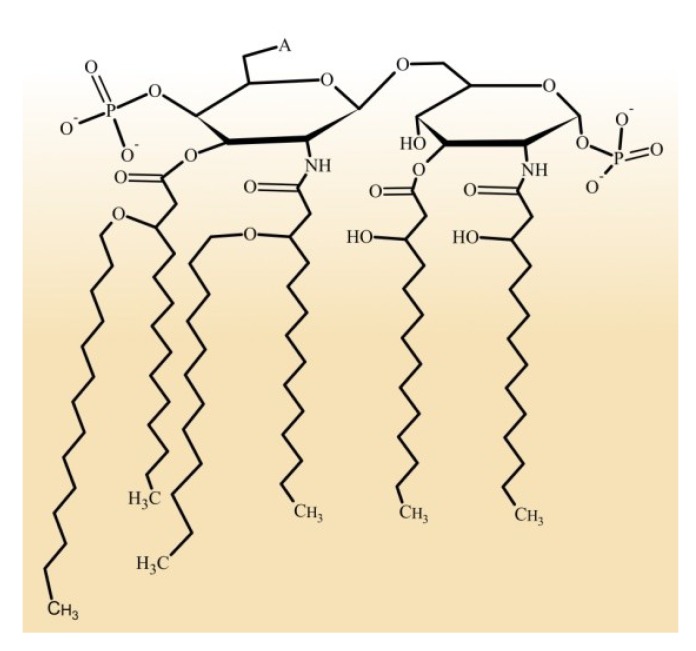
A representative structure of lipid A from *Escherichia coli*. This lipid A contains two phosphate groups in each glycosaminoglycan at the C1 and C4′ positions, as well as six fatty acid chains in the amide and ester linkage. A indicates the core attachment position.

Lipid A (also known as endotoxin) is a unique and distinctive hydrophobic segment that anchors the LPS to the membrane (Figure 2). It is a glucosamine-based saccharolipid [12] that constitutes the outer leaflet of the external membranes of most Gram-negative bacteria [13,14]. With a few exceptions [15,16], the lipid A and Kdo domains are important for the proper growth of bacteria [17,18,19]. These complex glycoforms are dispensable for growth but are required for the integrity of bacteria and to protect them from complement-mediated lysis and antibiotics. Virulence depends on the core and O-antigen domains, which, consequently, are present in most clinical isolates.

Structural characterization of LPS is quite challenging owing to its complexity and heterogeneous nature. This process involves a series of extraction, refinement, and fragmentation steps, supported by rigorous chemical analysis such as mass spectrometry (MS), matrix-assisted laser desorption/ionization (MALDI)-MS, gas chromatography, and nuclear magnetic resonance (NMR) spectroscopy to obtain the fine details of the complete LPS structure [20,21].

**Figure 3:**
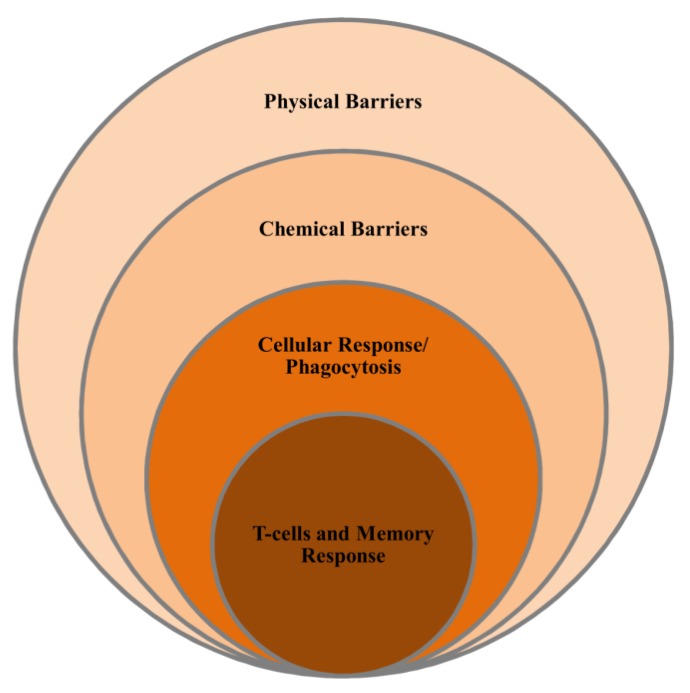
General overview of the defense system of marine organisms. This system is present in all organisms to varying degrees. In lower organisms, a few innate immunity structures such as protective skin and antibacterial peptides are sufficient to defend all challenges, whereas in higher animals, sophisticated and elaborate mechanisms have evolved to defend the host against a variety of threats. This includes specific responses such as CD8+ T cells, phagocytosis, immunoglobulins, a variety of chemokines, and the complement system.

The principal components used to detect a bacterial threat in animals are those of the innate immune system, including the Toll-like receptor (TLR) family, which is involved in sensing various components of bacteria [22]. TLR4 is dedicated to detecting LPS and mounts an immune response to counter a bacterial attack (Figure 3). In marine organisms, from sponges to higher vertebrates, homologs of TLR4 provide protection from bacteria. Apart from TLR4 and its homologs, bacteria have to cross certain barriers in order to potentially infect an animal. These barriers include physical structures as well as the immune system itself. To protect against such pathological invaders, marine organisms have evolved several rudimentary mechanisms capable of protecting the animal against a wide variety of pathogens. These measures include mucus supplemented with antimicrobial peptides and lysozyme, the complementary system, immunoglobulin, and other cytokines [23].

In this review, we discuss the different features of LPS that are displayed by various marine Gram-negative bacteria, the immune response mounted by infected animals, and several diseases that result from the host-pathogen interaction. Furthermore, we discuss the potential of LPS for treating diseases as an immune adjuvant.

## 2. Ecological Diversity of Marine Bacteria

The marine realm represents the largest inhabitable biosphere where living organisms flourish, especially microbes. Marine microorganisms thrive everywhere in the sea, from the surface water to the deep sea and from coastal to offshore regions, in both general areas and in specialized niches [24]. Marine life is broadly classified into six main groups: animals, plants, chromists, fungi, protozoa, and bacteria. All groups are heterogeneous in nature and are interdependent for survival. Among these groups, bacteria are the smallest in size and are capable of infecting other forms of life. Bacteria can live symbiotically or act pathogenically. Here, we will highlight the bacterial diversity and some potential diseases that afflict marine life [25].

In 2006, Cervino *et al.* [26] reported that *Ianthella basta*, a marine sponge inhabiting Kimbe Bay, Papua New Guinea, faced high mortality. The authors attributed the high mortality rate to five bacterial species that were prevalent in diseased sponges but absent from healthy sponges. Sequence analysis of 16S rRNA from these bacteria revealed that the culprits were two *Bacillus* species and three *Pseudomonas* species.

Although fish possess a robust immune system, they can nevertheless succumb to different bacterial diseases. Vibriosis is an important disease caused by various species of Vibrionaceae, including *Listonella (Vibrio) anguillarum* [27], *V. ordalii* [28], *V. salmonicida* [29], and *V. vulnificus* biotype 2 [30]. These species cause severe and economically important diseases in marine life. In addition, winter ulcer, caused by *Moritella viscosa* (formally known as *Vibrio viscosus*) and other members of this genus, is a significant problem for oceanic organisms [31].

*Photobacterium damselae* is associated with pasteurellosis, which is damaging to white perch and striped bass [32]. Different *Pseudomonas* species, including *P. chlororaphis*, *P. anguilliseptica*, *P. fluorescens*, *P. putida*, and *P. plecoglossicida*, have been isolated from deceased fish. All of these species cause diseases with various severities; *P. anguilliseptica* is considered a highly dangerous fish pathogen [33,34]. Furunculosis in salmonids, caused by *Aeromonas salmonicida* subsp. *salmonicida* in fresh and marine water, is an important cause of economic havoc. This bacterium is widely distributed and also infects non-salmonid fish species [35,36]. The Brucellaceae family of bacteria is also widespread in marine mammals and causes brucellosis. *Brucella ceti* and *B. pinnipedialis* have been isolated from cetaceans and seals, respectively [37]. *Tenacibaculum maritimum* causes flexibacteriosis in cultured and wild fish around the globe [38,39]. Mycobacterium is a notorious bacterial family that causes mycobacteriosis or fish tuberculosis. Mycobacteriosis is a sub-acute to chronic disease that can affect nearly 200 marine and freshwater species. The important species of this family that are associated with fish tuberculosis include *M. marinum*, *M. scrofulaceum*, *M. chelonae*, *M. smegmatis*, *M. abscessus*, *M. fortuitum*, *M. neonarum*, *M. simiae*, *M. poriferae*, and *M. triplex* [40,41,42,43,44,45,46,47,48,49]. *Piscirickettsia salmonis*, a non-motile Gram-negative bacterium that is responsible for high rates of mortality in marine animals, causes the disease in salmonids known as Piscirickettsiosis [50]. This is an obligatory intracellular bacterium that was initially reported in 1989 when infected Coho salmon were analyzed [51] after 90% mortality was observed in infected animals.

*Campylobacter* species (*C. insulaenigrae* and *C. lari*) were recently identified to infect various seals (*Arctocephalus gazella* and *Leptonychotes weddellii*). This was the first report of isolation of *Campylobacter* from seals of the Antarctic region [52]. *Salmonella enterica*, a contaminant isolated from humans to marine life, is a threat to a wide range of marine birds and mammals, including pinnipeds [53,54,55] and sea lions [56]. In addition, different *Campylobacter* and *Salmonella* species have been detected in northern elephant seals that never encounter water and in seals whose habitats are limited to the coast. Stranded seals showed a high prevalence of pathogenic bacteria that were likely of a terrestrial origin [54].

Bacteria belonging to the family Pasteurellaceae have been recovered from marine mammals using various methods of genetic, morphological, and evolutionary analyses. These isolates have been classified as related to *Pasteurella canis* found in sea lions, *P. stomatis*, *Bisgaardia genomospecies* 1, and *Bisgaardia hudsonensis* infecting harbor seals and grey seals, and *Otariodibacter oris* isolated from northern fur seals, walruses, and California and Steller sea lions [57,58]. These species can exist either in commensal or pathogenic relationships.

*Actinobacillus delphinicola* is frequently isolated from various cetacean species, whereas another species of this genus, *A. scotiae*, is found to infect porpoises, although rarely, in which it induced septicemia in three carcasses [59]. *Bordetella bronchiseptica* is an opportunistic bacterium that causes secondary infection in seals; epidemic reports indicated that all bacteria belong to one ribotype [60,61]. These bacterial species have caused severe damage to marine life during recent outbreaks.

## 3. Structural Organization of LPS

The general architecture of LPS is largely conserved and is readily distinguishable into three distinct forms, which each show variation in structure, functional groups, and bonding patterns [62]. With the advent of purification and structural determination procedures for LPS developed in the 1950s, particularly the classic phenol-water isolation technique developed by Otto Westphal, Otto Lüderitz, and Fritz Bister [63], it has been possible to identify structural determinants of various bacterial LPS.

Structural characterization of each region of LPS has been performed independently to ensure the determination of the correct bonding pattern and number of atoms, which demands careful handling. In this review, LPS structures from several prominent bacterial species are presented as background information. A more detailed description is available elsewhere [64,65].

### 3.1. Aeromonas

**Figure 4:**
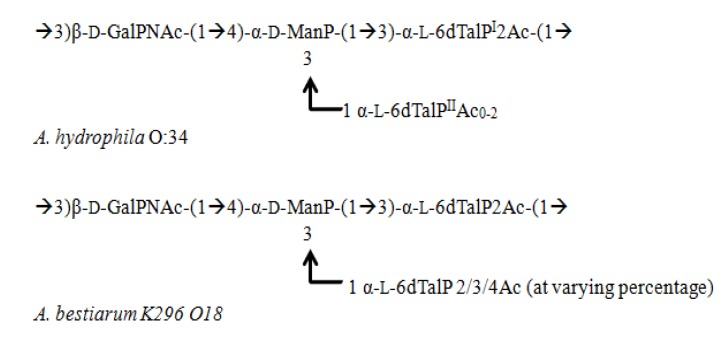
Structural representation of OPSes from *Aeromonas* genus.

The O-specific polysaccharide from *Aeromonas* species (*A. bestiarum* strain K296, serotype O18 [66] and *A. hydrophila* AH-3 [67]) have been elucidated combing NMR spectroscopy, MS, and chemical analyses. *Aeromonas* species are commonly present in various environmental conditions and are potential pathogens in fish and other marine organisms [68,69]. The aforementioned analyses revealed a similar structure in both species with slight variation in acetylation patterns. In the case of *A. bestiarum* K296, MS analysis unveiled that hexa-acylated or tetra-acylated lipid A is abundant in this species within the canonical backbone. This backbone consists of two saccharide molecules linked in a β(1→6) fashion, and the 1 and 4′ positions of each saccharide are substituted with phosphates with an AraN residue, as a non-stoichiometric substituent, and a core oligosaccharide composed of Kdo1Hep6Hex1HexN1P1. OPSes from both species are given below (Figure 4).

The glycosylation pattern and sugar composition in the OPS structures are similar in both species and follow a similar architecture of a tetrasaccharide unit with two 6-deoxy-l-talose (6dTalp), one ManP, and one GalpNAc residues. However, there is slight variation in their acetylation patterns in that 2-O acetylation was observed in the branched region of 6dTalp, while O-2 and O-4 (or O-3) were acetylated in the terminal 6dTalp region in *A. bestiarum* K296, whereas only O-2 acetylation was observed in *A. hydrophila* O34 [66,67]. Furthermore, biochemical analysis confirmed that serotypes O18 and O34 of *A. bestiarum* K296 trigger an antibody response, which shares the same epitopes in the LPS.

### 3.2. Pseudoalteromonas

The genus *Pseudoalteromonas* is a diverse group of marine pathogens that are Gram-negative, aerobic, and non-fermentative and require seawater for growth [70,71]. Studies have revealed that most of their O-antigens are acidic with a non-sugar substituent. The structure of colitose, a 6-C monosaccharide present in the O-antigen of *P. carrageenovora* [72], was determined by an initial O-deacylation of the LPS that yielded a soluble polysaccharide, followed by simple determination of the colitose residues. Colitose can either be attached at the terminus or located within the oligosaccharide motif.

Various approaches have recently been used for the characterization of core-OS from *P. carrageenovora* [72], which were found to be a mixture of three glycoforms with variable numbers of saccharide units and phosphorylation patterns. They display an extraordinary accumulation of phosphate groups in the core region, constituting a highly charged region.

**Figure 5:**
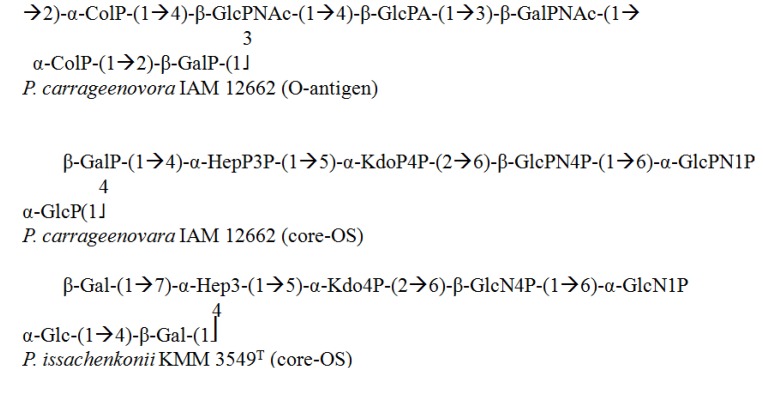
Structural representation of saccharide moieties from *Pseudoalteromonas* genus.

Similar to the aforementioned architecture, the chemical structure of the core region from *P. issachenkonii*’s LPS has also been evaluated [73]. This microorganism is equipped with various enzymes (alginases, fucoidanases, β-xylosidases, pullulanases, laminaranases, β-galactosidases, agarases, β-glucosidases, and β-*N*-acetylglucosaminidases) that can lyse bacteria, cleave proteins, degrade algal polysaccharides, and cause hemolysis. This core-OS structure was determined using alkaline deacylation accompanied by NMR and MALDI-MS, and the constituents were found to be three glycoforms with variable glycosylation and phosphorylation patterns (Figure 5).

It is pertinent to mention that glycoform 3 has three phosphates on Kdo and lipid A and lacks a phosphate on the O-3 of the heptose unit. Furthermore, in glycoforms 2 and 3 from *P. issachenkonii*, a heptose with 4,7-di-substitution is evident in the core region, which is a unique configuration. In addition, the core structure of LPS from *P. haloplanktis* TAC 125 has been reported [74]. *P. haloplanktis* also has a characteristic carbohydrate skeleton similar to *P. carrageenovora* and *P. issachenkonii,* which appears to be a common feature of the *Pseudoalteromonas* genus.

The lipid A structure has been reported for three different species of the genus *Pseudoalteromonas* [75,76], including *P. issachenkonii* [73]. When the lipid A structure of *P. issachenkonii* KMM 3549T was analyzed, it was noted that in contrast to *P. haloplanktis*, the prominent entity is a tetra-acyl group rather than a penta-acyl group [73]. This tetra-acyl moiety, composed of three C12:0(3-OH) residues and one C12:0 residue, has been previously observed. Penta-acyl and tri-acyl structures have also been detected. A similar fatty acid chain and substitution profile in lipid A was noted from *P. carrageenovora* IAM 12662T. The presence of rare acyl components may also be attributed to the environment from which these species have been isolated. The prominent features of lipid A from various bacteria are provided in Table 1.

**Table 1:** Acylation profile of different lipid A species from Gram-negative marine bacteria.

Bacterium	Type and linkage of acyl substituent				
	GlcN II ^a^		GlcN I		Reference
	3′	2′	3	2	
*Pseudoalteromonas carrageenovora*	10:0(3-OH) ^b^	12:0(3-O-12:0)	-	12:0(3-OH)	[64]
*Pseudoalteromonas issachenkonii*	10:0(3-OH) ^b^	12:0(3-O-12:0)	-	12:0(3-OH)	[73]
*Alteromonas addita*	12:0(3-OH)	14:0(3-O-12:0)	10:0(3-OH)	14:0(3-OH)	[77]
*Alteromonas macleodii*	12:0(3-OH) ^b^	12:0(3-O-12:0) ^c^	-	12:0(3-OH)	[78]
*Shewanella pacifica*	13:0(3-O-13:0)	13:0(3-O-13:0)	13:0(3-OH)	13:0(3-OH)	[10]
*Arenibacter certesii*	15:0(3-OH)	15:0(3-O-15:0)	15:0(3-OH)	15:0(3-OH)	[11]

^a^ 2,3-diamino-2,3-dideoxy-d-glucopyranose (DAG) in *Arenibacter certesii* KMM 3941T; ^b^ alternative substitution at position C3; ^c^ interchangeable.

### 3.3. Shewanella

**Figure 6:**
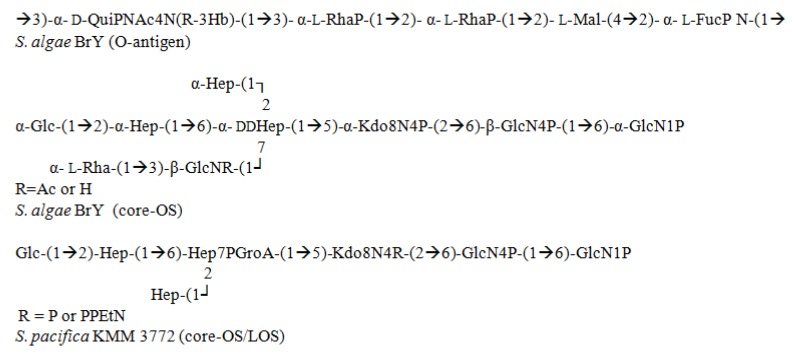
Structural representation of O-antigen and Core-OS from *Shewanella* genus.

The *Shewanella* genus encompasses more than 20 described species that have a widespread habitat and interaction mode. Free-living and symbiotic forms have been recovered from different marine species, including algae and fish. *S. putrefaciens* and *S. algae* are pathogens in humans and marine life that cause bacteremia and septic shock. Numerous OPSes have been characterized from members of this species to date. These OPSes contain acidic polysaccharides as well as acidic non-carbohydrate substituents, including malic acid, as reported for *S. algae* BrY [79] (Figure 6), or the specific monosaccharide Shewanellose [(2-acetamido-2,6-dideoxy-4-*C*-(3′-carboxamide-2′,2′-dihydroxypropyl)-d-galactose], a novel C-branched sugar. This particular sugar was reported for the first time from *S. putrefaciens* A6, accompanying an 8-epilegionamminic acid derivative [80].

The presence of Kdo8N instead of Kdo has been confirmed in the core-OS from other bacterial species [9,10,81]. The structural similarities in the core-OS suggest a modification in the structure from *S. algae* BrY that results in various conformations.

By employing various chemicals and analytical techniques, the core-OS structure from *S. pacifica* KMM 3772, 3605, and 3601 was determined [10]. As mentioned above, in *S. pacifica*, Kdo8N is the principal residue that replaces Kdo. Moreover, this Kdo8N residue associates with an atypical heptose residue (d,d-Hep) at the C-5 position, which is supplemented with a non-carbohydrate moiety bonded with a phosphodiester linkage, as characterized from *S. oneidensis* MR-1 LPS. Another feature that contributes to the overall ionic character of the LPS molecule from *S. pacifica* is the existence of a phosphodiester-linked glyceric acid to the d,d-Hep residue. Even though this is a common metabolic intermediate of Gram-negative bacteria, it is rarely observed in core regions.

The acylation profile in the lipid A of *S. pacifica* KMM 3772 has been analyzed using MS data [10]. The major acyl found was hexa-acyl, showing both types of bonding patterns: amide and ester with primary fatty acids, (*R*)-3-hydroxy-tridecanoic acid [13:0(3-OH)]. At GlcN II, both primary fatty acids also have a tridecanoic acid [13:0(3-OH)] appendage.

### 3.4. Cytophaga-Flavobacterium-Flexibacter Phylum

**Figure 7:**
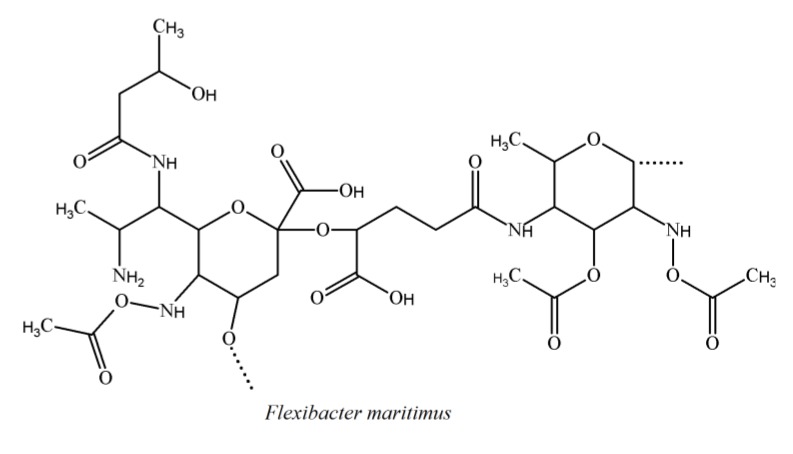
The repeating structural unit of *Flexibacter maritimus* LPS.

The structural diversity of LPS has also been evaluated from the fish pathogen *Flexibacter maritimus* [82]. The structure of O-antigen from *F. maritimus* has been elucidated by extensive spectroscopy analysis of the polysaccharide and its methanolysis product. The data revealed the occurrence of higher monosaccharides (pentadeoxynonulopyranosonic acid decorated with various groups), together with extensively substituted trideoxy-β-glucose (QuiNAc3Ac4NAcyl). The repeating unit is shown below (Figure 7).

This repeating unit bonds to the (*S*)-2-hydroxyglutaric acid residue. This O-antigen is also present in another fish pathogen, *Flavobacterium psychrophilum*, which has similar components such as O-glycosylated amide-linked (R)-malic acid [83,84]. *F. maritimus* and *F. psychrophilum* belong to the same phylum, which indicates a conserved structure in this lineage. *Cellulophaga fucicola*, which belongs to the Flavobacteriaceae family, was found to have a trisaccharide-repeating unit when hydrolyzed by using a mild acid treatment. This trisaccharide unit is supplemented with pseudaminic acid [84].

### 3.5. Alteromonas

**Figure 8:**
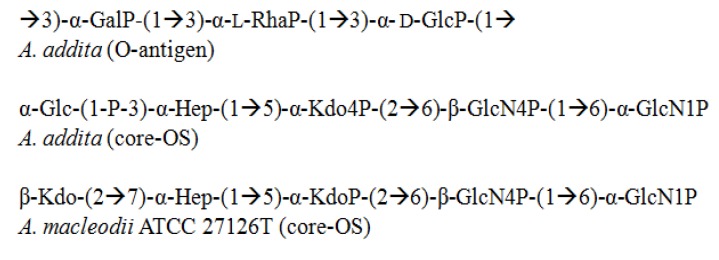
Structural representation of O-antigen and core-OS from *Alteromonas* genus.

The *Alteromonas* genus*,* initially classified by Baumann *et al.* [85], was reclassified into *Pseudoalteromonas* and *Alteromonas* [86]. The new *Alteromonas* genus comprises several species, including *A. macleodii*, *A. marina*, *A. stellipolaris*, *A. litorea*, and *A. addita*. Studies have been carried out to investigate the structural components of the LPS from *A. macleodii* ATCC 27126T [78] and *A. addita* KMM 3600T [77] (Figure 8). In both species, bacteria produce R-LPS with short chain lengths, carrying a very negative charge. The reduced saccharide structure thus fits within the definition of OS. In the core-OS from *A. macleodii* ATCC 27126T, Kdo is the component present in β-configuration that imparts the negative charge, along with some other residues. This is a very rare feature for LPS in general, and is more often present in polysaccharide capsules [87].

*A. addita*, a new addition to the genus, was first isolated from seawater samples from different parts of a sea near Japan that were collected to study the effects of radiation on free-living microbial colonies [88]. The structural determination of the core-OS of the lipooligosaccharide (LOS) from *A. addita* was accomplished by using 1H-, 13C-, and 31P-NMR spectroscopy and MALDI-MS. Initially, the structure was completely deacylated and subjected to selective mild O-deacylation to isolate the core region [77]. This latter enabled the detection of a glucose moiety linked to a heptose residue through a phosphodiester bond.

Furthermore, the lipid A structures of *A. macleodii* ATCC 27126T [78] and *A. addita* KMM 3600T [77] have been determined, along with structural elucidation of the O-antigen and core-OS by using MALDI-MS. The analysis of the whole lipid A, treated with a weak base from *A. macleodii* ATCC 27126T, produced a mixture of tri-, tetra-, and penta-acyl species, of which tetra-acyl was prominent. In particular, lipid A with a 5-chain was supplemented with two C12:0(3-OH) residues in an amide bond and two C12:0(3-OH) residues in an ester bond directly linked to saccharide units, while one was attached as an appendage (C12:0) to form a secondary fatty acid at C2'. Tetra- and tri-acyl lipid A are deficient in one or two ester-linked 12:0(3-OH) residues [78].

When lipid A from *A. addita* was analyzed using a similar approach [77], it appeared that a penta-acyl carried lipid A, in which a [3 + 2] distribution was observed on the GlcN. In this bacterium, the dominant species displayed two chains of (*R*)-3-hydroxytetradecanoic acid [14:0(3-OH)] as amide substituents. In the case of GlcN I, this residue was replaced by an (*R*)-3-hydroxy-tridecanoic acid [13:0(3-OH)]. Acylation by a 14:0 or a 12:0 residue was secondary at GlcN II, whereas there was a 10:0(3-OH) fatty acid in ester linkage at the C3 position.

### 3.6. Arenibacter

*Arenibacter* is a genus of Gram-negative marine bacteria that are non-motile, require O_2_ for growth, are heterotrophic, and have dark-orange pigmentation, and are classified in the Cytophaga-Flavobacterium-Bacteroides phylum [89]. The structural organization of lipid A from *Arenibacter certesii* KMM 3941T indicates penta-acyl as the dominant form [11]. The backbone of this glycolipid consists of a 2,3-diamino-2,3-dideoxy-d-glucopyranose (DAG)-GlcpN, linked in a β-(1→6) fashion, with canonical phosphorylation sites at C1 and C4′. The fatty acid chains are of pentadecanoic form in primary substitution with the 3′-OH, while there is only one in secondary substitution. Based on spectrometry data, a 15:0 residue is indicated in an ester linkage on either primary amide substituent of the incompletely fragmented lipid A. *Chryseobacterium scophtalmum* CIP 104199T, which belongs to the same phylum as *A. certesii*, shows a highly unusual lipid A architecture [90]. This lipid A possesses a monosaccharide unit with its C1 phosphorylated and supplemented with one (*R*)-3-hydroxy-15-methylhexadecanoic and one (*R*)-3-hydroxy-13-methyltetradecanoic residue as primary amide and ester substituents, respectively. This is a close relative of lipid X, which is a lipid A biosynthetic precursor in *Escherichia coli.* This may be related to the absence of a phospholipid (PG) in the membranes of *C. scophtalmum*; indeed, bacteria deficient in PG tend to accumulate lipid X [91,92,93].

**Figure 9:**
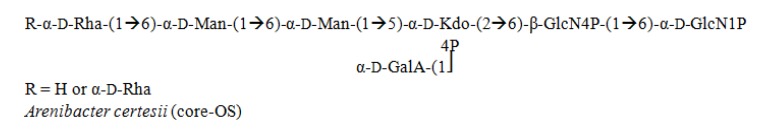
Structural representation of core-OS moiety from *Arenibacter certesii*.

The presence of a GalA residue imparts a negative charge to increase the total charge per unit of the molecule. These negative charges help in building ionic bridges between LPS and cations in the environment to further confer stability to the cell wall of Gram-negative bacteria [94] (Figure 9).

In addition to the aforementioned lipid A structures, initial characterization has been performed on the fatty acid components (but not their arrangement) from the glycolipid moieties in other *Pseudoalteromonas* species [95]. In all cases, there is similarity between the fatty acid composition and that of the known lipid A molecules, with a preference for short-chain fatty acids.

## 4. Marine Immune System

**Figure 10:**
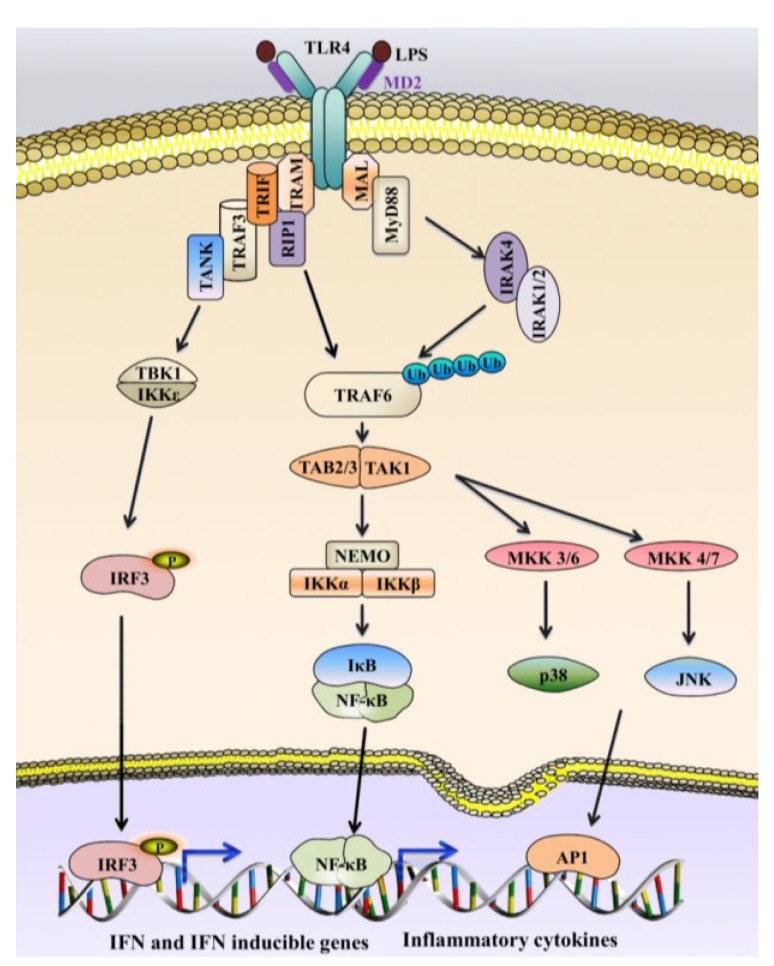
General overview of TLR4 signaling in humans. TLR4 is activated by LPS, which in turn triggers both MyD88-dependent and MyD88-independent TRIF-dependent pathways. Signaling through the MyD88-dependent pathway is responsible for early phase nuclear factor (NF)-κB and mitogen-activated protein kinase (MAPK) activation that facilitates the induction of pro-inflammatory cytokines. The TRIF-dependent pathway activates IRF3, which culminates in the induction of IFN-β- and IFN-inducible genes. This pathway also helps in secondary NF-κB and MAPK activation that reinforces the defense response.

Marine organisms, from sponges to vertebrates, possess either an innate immune system or a rudimentary adaptive system and live in an adverse environment that is characterized by high pressure and an abundance of microbes (Figure 10). Successful survival in such an environment is solely based on an efficient and strong innate immune system to fight against potential invaders [96]. In the past few years, with advancements in techniques, the immune system repository has been extended to define the specific immune molecules that are vital for these organisms to survive. Although modern technologies boost such exploration, understanding the marine immune system is still in its infancy, and little is known about host-pathogen interactions.

AP1, activator protein 1; IFN, interferon; IKK, inhibitor of nuclear factor-κB (IκB)-kinase; IRAK, IL-1 receptor-associated kinase; IRF3, interferon regulatory factor 3; IκB, inhibitor of κB; JNK, c-Jun *N*-terminal kinase; LPS, lipopolysaccharide; MAL, MyD88 adaptor-like; MD2, myeloid differentiation factor 2; MKK, MAPK kinase; MyD88, myeloid differentiation protein 88; NEMO, NF-κB essential modulator; NF-κB, nuclear factor κB; p38, protein 38; RIP1, receptor interacting protein 1; TAB3, transforming growth factor-β-activated kinase 1/MAP3K7-binding protein 3; TAK1, transforming growth factor-activated kinase 1; TANK, TRAF-family member-associated NF-κB activator; TBK, TANK-binding kinase; TLR, Toll-like receptor; TRAF3/6, tumor necrosis factor (TNF) receptor-associated factor 3/6; TRAM, TRIF-related adaptor molecule; TRIF, TIR-domain-containing adapter-inducing interferon-β factor.

### 4.1. Non-Specific Immunity

Non-specific immunity is the principal defense mechanism in lower vertebrates and fish that helps protect the animals from pathogens, a result of the rudimentary adaptive immune system, poikilothermic nature, slow proliferation and lymphocyte memory, and limited antibody diversity that is characteristic in these organisms [97]. The innate response comprises three parts: the physical barrier, the humoral response, and cellular components. The physical barrier, which includes epithelial and mucous membranes, is an extremely important disease barrier because it is continuously in contact with harmful agents [98].

(1) Physical barriers such as the skin, mucus, flakes, and gills are the first barriers against pathogens [99,100,101]. These not only contain important compounds, antibacterial peptides, and immunoglobulin M (IgM) that hinder pathogen entry [102,103,104,105,106], but also maintain resistance against thickening and cellular hyperplasia [107].

(2) In marine organisms, a homolog of natural killer cells mounts a non-specific response to remove various pathogens such as parasites and canonical targets of NK (natural killer) cells in higher vertebrates [108,109].

(3) Different studies regarding antimicrobial peptides in fish [110] have demonstrated their physiologically important role in host defense against microbes. These polypeptides are able to disintegrate cell walls [101,111,112].

(4) Phagocytosis is a temperature-independent and integral process in poikilothermic animals that is carried out by macrophages and neutrophils [113,114,115,116]. The principal mechanism used to degrade bacteria is by generating nitric oxide and reactive oxygen species (ROS) that act as effective antibacterial agents [116,117].

(5) With a poor understanding of the complement system, except for the sequence of mannose-binding lectin protease, some studies regard the alternative complement pathway as a valuable asset to marine animals [118,119,120,121]. Alternative complement activities have been well documented in different species [121,122,123,124]. During bacterial invasion, the complement system is activated by LPS and culminates in C5a factor production, which helps to activate macrophages and neutrophils, resulting in the phagocytosis of pathogenic bacteria [122].

(6) Several studies in different fish species have pointed to the presence and central role of cytokines and chemokines, including tumor necrosis factor (TNF)-α and TNF-β, interferon (IFN), interleukin (IL)-6, IL-1β, and colony-stimulating factors (CSFs), which activate a diverse class of immune and tissue cells [125,126,127,128,129,130,131,132,133,134]. These various activating molecules boost phagocytosis and trigger cells to produce antiviral and antibacterial protein molecules that resist bacterial and viral insult through several cell-dependent and cell-independent mechanisms.

(7) Several proteins, including protease inhibitors, C-reactive protein (CRP), serum amyloid protein (SAA), and lysozyme (cell wall degrading enzyme), are present in various body fluids of higher marine animals [135,136,137]. These proteins are mainly associated with body homeostasis but are also involved in acute phase infection and inflammation [98]. The level of these proteins increases with tissue injury or infection, and the proteins are also involved in complement activation and removal of apoptotic cells [138,139,140]. In addition, lysozyme is widely distributed and degrades the cell wall of invading Gram-negative bacteria [141,142].

(8) In marine organisms, fish are capable of producing natural antibodies, even in the absence of antigens [143]. These antibodies play a key role in defense against a broad spectrum of pathogens and also provide adaptive immunity [97].

### 4.2. Specific Immunity

The adaptive immune response present in marine animals consists of a complex network of coordinated entities that respond against antigens, antibodies, and other effector cells. Invertebrates lack a specific response, whereas lower vertebrates exhibit a basic form.

(1) When considering the immunoglobulins, IgM is the predominant immunoglobulin in fish, existing predominantly as a tetramer with eight paratopes [144] and as a monomer in some organisms [145]. Both forms have the same binding affinities but differ in their capabilities to activate the complement system, which can be potently activated by a tetramer [146]. The second identified immunoglobulin is the IgD isotype, identified in catfish, which shows sequence similarity to mammalian IgD and is located adjacent to IgM [147]. These antibodies are prevalent in the skin, intestine, gill mucus, bile, and plasma [148,149,150,151].

(2) There is a large body of evidence that fish are capable of developing a memory response after challenge with an antigen [152,153]. It is pertinent to mention that two exposures are required for T-dependent antigens, whereas only one is sufficient for T-independent antigens. This behavior helps marine animals better cope with subsequent bacterial exposure.

(3) Apart from a humoral response, fish can demonstrate cell-based cytotoxicity reactions. However, the cells participating in cell-mediated cytotoxicity are difficult to define owing to the lack of suitable tools and molecular recognition [117]. To combat viral infection, there is a mechanism similar to the CD8+ cytotoxic T lymphocyte response that is critical to protect the animal from viral challenge. There are sequence similarities in major histocompatibility (MHC) class I and CD3+ T cells between higher vertebrates and fish that imply similar mechanisms [117]. Similar to other vertebrates, different fish species attain immunocompetency early in development. For instance, T cells appear in sea bass (*Dicentrarchus labrax* L.) during larval development [154], and mRNA of T cells appears one week after fertilization [120]. CD4 has recently been discovered in teleosts, which helps to extend the repertoire of immune cells [155].

## 5. LPS Detection and Marine Immune Response

The marine immune response toward pathogenic challenge is poorly understood, but available data indicates that marine animals are well equipped with components that can defend against diverse potential intruders [96]. Recently, with the advent of modern techniques, knowledge of the immune system repertoire in lower vertebrates and invertebrates has been extended.

During evolution, TLR4 developed a novel function to detect bacterial LPS [156]. Homologs of TLR4 have been reported in invertebrates such as sponges [157], fish (zebrafish have two types of TLR4 [158]), and other higher vertebrates. The specificity of TLR4 to detect LPS has been extensively utilized to stimulate the immune system in various studies [159,160]. It is likely that there are alternative methods for detecting LPS in other species in which a TLR4 homolog has not yet been identified, or the possibility remains that contamination in LPS preparation has caused the contrasting results [161]. However, scarce data is currently available to resolve this discrepancy.

Sponges are filter feeders that are the earliest-evolved members of the phylum Porifera. Living in a marine environment and possessing their unique type of feeding pattern continuously exposes sponges to marine microorganisms. Wiens *et al.* [157] reported that the sponge *Suberites domuncula* was able to recognize LPS from Gram-negative bacteria. The protein that can detect LPS is referred to as the lipopolysaccharide-interacting protein, and it is present on the cell surface. This protein can dimerize and interact with the sponge myeloid differentiation protein 88 (MyD88). Two domains have been identified in sponge MyD88, a Toll/interleukin-1 receptor domain and a death domain. Further experiments revealed induced expression of MyD88 and no alteration in the levels of LPS-interacting protein after LPS detection. Downstream in this pathway, there is another inducible protein, macrophage-expressed protein, which possesses biological activity and eliminates invading Gram-negative bacteria; Gram-positive bacteria are insensitive to this protein. This study unveiled that the innate immune system in *S. domuncula* includes a pattern recognition receptor (LPS-interacting protein), an adaptor molecule, MyD88, and an executing molecule, macrophage-expressed protein [157].

Recently, it was demonstrated that *Scylla paramamosain* could mount cellular and non-specific responses when challenged with LPS. Exposure to LPS triggered many immune-related processes such as induced production of lysozyme, increased phagocytosis, antibacterial activity, and phenoloxidase, as well as production of superoxide and nitric oxide during the treatment period. There was a significant correlation between the immune response and LPS challenge. Moreover, it was observed that increased ROS generation was accompanied by an increase in antioxidants to monitor the oxyradicals generated. This study delineated the various immune-related changes in crab hemocytes that occur to cope with infection; such responses could be modulated for therapeutic purposes [162].

In another study, an immune mechanism was deciphered from the corals *Porites astreoides*, *Montastraea faveolata*, and *Stephanocoenia intersepta*, which are vulnerable to various diseases and are susceptible to bleaching. In addition to the effect of temperature on the immune response, an immune stimulator LPS was also studied. The immune parameters that have been studied include bactericidal activity, melanin concentration, prophenoloxidase (PPO) activity, and catalase, peroxidase, and fluorescent protein (FP) concentrations. All three species responded differently to LPS, depending on the experimental conditions. The disease-susceptible species, *M. faveolata*, showed substantially lower bactericidal activity and melanin concentration, whereas the disease-resistant species, *P. astreoides*, displayed induced expression of enzymatic antioxidants after exposure to LPS. *S. intersepta*, when challenged with LPS, exhibited increased activity of PPO, decreased bactericidal activity, and reduced expression of FPs. This study unveiled the immune mechanisms of corals challenged with pathogen-associated molecular patterns (PAMPs) that lead to increased levels of molecules involved in host defense [163].

A new aspect of the marine immune system has recently been unveiled, indicating that TLR4 does not recognize LPS, and that LPS might even antagonize TLR signaling. This study supports the notion that amphibians and fish are insensitive to LPS toxic shock. This report included three different fish that have an unknown TLR or are lacking a TLR4 ortholog (gilthead seabream and spotted green pufferfish) or possess the TLR4 ortholog (zebrafish). Experiments showed that LPS signaling was independent of TLR4 and MD-2, and that zebrafish TLR4 negatively regulated MyD88-dependent LPS signaling. These are the underlying mechanisms to explain these species’ resistance to endotoxic shock [164].

A variety of TLR homologs are present in fish, where they serve to protect against pathogens. However, the molecules involved in LPS recognition have not been described in detail. Several fish species show pronounced inflammation, cytokine release, and other physiological effects in response to LPS [165], providing insight into the availability of defense mechanisms against endotoxins.

Identification of an MD2 homolog in marine organisms has been a challenge. For instance, the sequence comparison of TLR4 from zebrafish to that of humans and mice revealed striking differences that point to the absence of MD2 [158]. In zebrafish, GFP-tagged TLR4a/b was shown to translocate to the membrane surface, which further strengthens the hypothesis that zebrafish is either lacking MD2 or has devised a unique mechanism to propagate the signal [158].

Moreover, the phylogeny of MD2 supports the notion that this molecule has evolved relatively recently to facilitate the LPS response. This co-receptor molecule is less conserved in animals, and any significantly related sequence is lacking in any of the fish expressed sequence tag databases. Furthermore, this gene is fragmented by numerous introns that render *in silico* identification very difficult. Consequently, it is difficult to identify this sequence from the genomic database [166].

Finally, it is debatable whether CD14/MD2/TLR4-mediated responses exist in fish. Although TLR4 is expressed in lower animals and might still be involved in the immune response to LPS, the specific mechanism of its involvement might differ between higher and lower vertebrates, including fish. It is possible that the ability of TLR4 to initiate a rapid inflammatory response to even a minute amount of endotoxin evolved later in evolution to protect higher vertebrates; this property might indirectly depend on the evolution of accessory molecules (CD14 and MD2). In piscines, the alternative signaling pathways may be of importance to stimulate the leukocytes by LPS [166].

## 6. Structure-Activity Relationship of LPS

In LPS pathology, O-antigen is mainly involved in activation of the humoral response, whereas lipid A is principally involved in stimulating the inflammatory response. In lower animals, the adaptive immune system is of a rudimentary form that involves the production of a few antibodies that helps to defend the host, while the innate immune system is reasonably strong and can effectively eradicate the invading microbes. Many structural modifications in LPS structure, particularly in lipid A, can circumvent the host immune response. The details regarding the structure-activity relationship of LPS have been reviewed elsewhere [167].

Lipid A is unambiguously a physiologically active component of endotoxin [168,169,170,171]. To correlate the structural features of lipid A to its endotoxin activity, various experiments have been performed involving different analogs lacking important functional groups. The monosaccharide form of lipid A is the least active (<10^7^-fold decrease in activity) [172]. The removal of phosphate groups, either one or both, also results in a reduction in biological activity [168]. Moreover, altering the hydrophobic region, either by adding one fatty acid chain to make hepta-acyl lipid A or by removing one chain to result in a penta-acyl chain, also hampers the inflammatory response. Lipid A with a four-acyl chain is unable to induce inflammatory mediators in almost all cases. The length of the acyl chain also determines the biological activity, *i.e.*, increasing the length decreases the biological activity [173].

Recently, the release of inflammatory mediators were shown to depend on the LPS composition as well as on the specific cell types used in the study [174]. For example, lipid A from *E. coli* could activate both human and mouse cells, while *Salmonella* lipid A could activate only mouse cells [175]. Similarly, lipid A derived from *Leptospira interrogans* was unable to activate human THP-1 cells, but could trigger TNF-α release in RAW 264.7 cells [176]. These experiments revealed the species-specificity of endotoxins and may imply diversity in both the cellular detection of lipid A and their responses to various lipid A structures [177]. Furthermore, it is difficult to evaluate the absolute endotoxins properties of lipid A due to differences in cell types and the media used in its evaluation across studies.

## 7. Marine LPS as a Drug Candidate for Human Diseases

Research geared toward developing new therapeutic drugs is shifting into the exploration of marine-based compounds that can act either as therapeutics or as adjuvants in vaccine applications. Indeed, many recent reports have pointed to the potential value of marine-based compounds.

Warabi *et al.* [178] reported that Axinelloside A, a highly sulfated LPS, is a potent inhibitor of human telomerase (IC50 2.0 μg/mL) and could be isolated from a sponge, *Axinella infundibula*. Its chemical arrangement was deduced based on MS and two-dimensional NMR spectra, and its molecular weight, in a monoisotopic mass of sodium salt, is approximately 4780.4 g/mol [178]. This polysaccharide consists of a mixture of 12 sugars, including d-arabinose, scyllo-inositol, l-fucoses, and d-galactoses, together with an (*R*)-3-hydroxy-octadecanoic acid, 3(*E*)-2-hexadecenoic acids, and 19 sulfates. This compound shows potential for cancer treatment.

LPS and its lipid A from different marine bacteria have been studied for their therapeutic potential, including *Marinomonas communis*, *Marinomonas mediterranea*, and *Chryseobacterium indoltheticum*. Variable effects have been shown from each bacterial species. Importantly, no induction of TNF has been observed in peripheral human blood when lipid A of *C. indoltheticum* and LPS and/or lipid A from *M. communis* were used. Furthermore, LPS and lipid A from these species could inhibit the release of TNF-α in bacterial sepsis [179].

A recent study demonstrated that LPS from two species of Gram-negative marine bacteria of the genus *Pseudoalteromonas*, *P. luteoviolacea* and *P. ruthenica*, possess weak immunomodulatory activities compared to other Gram-negative bacteria. These LPSes also induced low levels of cytokines in murine bone marrow-derived dendritic cells and moderate up-regulation of CD40 and CD86 cell surface markers, as compared to *E. coli* LPS. Furthermore, these LPSes antagonized *E. coli* LPS and hindered IL-12 and IL-10 secretion. Consequently, these results demonstrated novel immunomodulators that could be used as therapeutic agents [180].

LPS could also be used as an adjuvant for inducing immunity against particular antigens. A hapten (NIP; 4-hydroxy-3-iodo-5-nitrophenylacetic acid) conjugated to chicken gamma globulin, a highly antigenic protein, induced a weak response in mice. By contrast, when this preparation was injected along with LPS, enormous levels of antibodies were generated against the specific antigen. This study provides a basis for further exploration of the adjuvant activity of highly toxic LPS in vaccine therapy [181].

## 8. Conclusions

Bacteria thriving in oceanic environments pose a threat for a variety of diseases that may hamper the growth of marine organisms as well as negatively impact the economy. Therefore, it is imperative to seek out ways to protect marine life from infection. Marine bacteria either reside in a commensalism or cause disease in marine organisms. Although the etiology and pathogenesis of different diseases vary, LPS as the integral part also influences host-pathogen interactions by triggering the innate immune system, which contributes to the outcome of infection.

The outer leaflet of the cell wall in Gram-negative bacteria harbors LPS, which shows diverse structural organization across species, although a common architecture is observed consisting of the O-antigen, core-OS, and lipid A regions. Within the same region, there are countless possibilities of bonding patterns as well as substitutions of small molecules. In LPS, lipid A interacts with the innate immune receptor and, owing to its diverse features, may either trigger or inhibit the downstream molecules of the immune-related pathway. The various features modulating this response as well as their influence have been reviewed. The core and O-antigen also show variation from simple forms, consisting of only a few types of residues, to varying degrees of complexity and charge distribution among different species. The structural elucidation of LPS remains a challenge due to its chemical composition. With the advent of new techniques and methods, more and more structures have been resolved.

In response to LPSes, marine organisms mount different immune responses that counter the bacterial threat by activating various defense systems. Therefore, these LPSes show potential as vaccine adjuvants to achieve a desired immune response or to activate or inhibit molecules targeting human diseases. New approaches in combining molecular and cellular techniques to decipher the mechanisms of bacterial diseases will provide a basis for novel mechanisms of immunology and host-pathogen interactions, as well as help to decipher the specific virulence factors in bacteria in addition to the evolutionary relationship between bacteria and the small molecules involved in bacterial sensing.
